# Not Alms, but a Friend

**Published:** 1887-12-10

**Authors:** 


### The Hospital

A Weekly Institutional Journal of

**Science, Medicine, and Philanthropy.**

*For Hospitals, Asylums, Medical Practitioners, Students, Nurses, Families, Parishes, Congregations, and the Charitable Public.*

Vol. III. No. 63.] [December 10, 1887.

### Not Alms, but a Friend.

The *Morning Post* in a leader takes up our proposed solution of the "unemployed" problem, and discusses it not unkindly. The scheme possesses such merit as appertains to long and honest consideration of the subject. That of course does not entitle it to acceptance, though perhaps it may be allowed to add weight to a plea for a fair and thoughtful hearing. Not a word need be said about the urgent and immediate importance of the question: we are all so convinced of it, that if any scheme were put before us of such a nature as to at once convince everyone of its fitness and practicability, everyone would immediately cry out for its adoption. But a little reflection will show that it is not possible for any man to propound a scheme so self-evidently perfect that every other man on hearing will immediately exclaim, like the monks with the "Jackdaw of Rheims," "That's it." Whatever be the nature of any plans which may be proposed, two things may be taken for granted regarding them: first, that they will not be perfect; and secondly, that they will not lack critics to point out their imperfections.

We suggest, urgently, that ours *is* a scheme, that it is more comprehensive in itself than any other now in the field, and that it is based on a wider and deeper knowledge of the subject than any which has appeared elsewhere. We do not say this in a spirit of boastful competition—far from it; but in order to urge that a practical, and not a merely speculative, consideration may be given to what is believed to be a practical proposal. The question, indeed, is of such a nature as to forbid mere academic treatment. A man who is a man at all does not too minutely examine a rope when he sees a fellow-being floundering helplessly in twenty feet of water. He flings the rope to him, such as it is, and in most cases brings him safely to shore.

There is some probability that a "test" beginning will be made in Paddington. The population of that large and mixed constituency have had the proposal put before them at a public meeting presided over by Lord Randolph Churchill, and it has been printed for private circulation. The parts of the scheme which, it is hoped, will be put into immediate operation there, consist of what may be called the "double registration"—the complete registration, on the one hand, of all the poor and unemployed; and the equally complete registration on the other, of all the resources and methods of charitable and other relief in the borough. The latter has been described as the "Relief Clearing House" part of the work; and it has been assumed that it must necessarily be "inquisitorial" in function. But that is not the idea at all. Its object is to deal with the whole of the distress at once and completely by means of friendly counsellors. It will enable us to avoid the Scylla of giving too much relief to the clamorous, on the one hand, and the Charybdis of giving too little, or none at all, to the shy and retiring, on the other. This is exactly what is needed. We must focus the distress clearly; and we must also focus, as clearly, every existing means of relieving it.

If the "double registration" scheme were carried out thoroughly in Paddington, and were found in practice to give all the precise and complete information which it seems capable of giving, then its immediate application to every other metropolitan borough would furnish us at once with an accurate bird's-eye view, a portrait in miniature, of the whole field of operations. With such a picture in his hand a skilful general could command the whole situation with the utmost ease. It would then be quite impossible for unrelieved distress to escape notice, and equally impossible for relieved distress to remove itself from the area of observation, and so to obtain relief again and again by mere migration.

This is the first part of the scheme. Where is the difficulty in it? There is no difficulty. The difficulty lies entirely with those who should feel impelled by every consideration of duty and of safety to carry it out. The prosperous classes and the governing classes ought not to allow a day to pass over before making a beginning.

The *Morning Post* does not disapprove of public relief works, nor of that particular work—the purification of the Thames—which we suggest as lying ready at hand. But it objects that such relief works would not settle the question "once and for all." Practical engineers, who have given much thought to the subject, are of opinion that such a scheme of Thames purification as has been proposed would provide employment for all the unemployed of London for ten years. That is looking pretty far ahead. But whilst we urge the Thames purification for the present emergency and for London, we urge with as much, or even more, earnestness the *home colonisation* proposal of Mr. Herbert Mills. Those people who have given two minutes' consideration to home colonisation, and who therefore know all about it, dismiss it with a wave of the hand as impracticable. But those who have a lifelong and practical acquaintance with agriculture know very well that Mr. Herbert Mills' ideas are in the highest degree reasonable and capable of being carried out in practice with the best results. In this, as in the case of the double registration scheme, the difficulty does not lie in the plan proposed at all—that is simplicity itself; the difficulty lies entirely with the prosperous and governing classes, who prefer folding their arms and lolling in their easy chairs, to rousing themselves to practical exertions in a cause which offers them no kind of immediate individual reward. The foolish people have not the intelligence to see that we are in the very midst of a national crisis, the magnitude of which none of us fully perceive; and that there is an immediate and most imperious necessity for work to be provided, whereby the hungry may be fed, and those who are made seditious by starvation may be made loyal by prompt help and rekindled hope.

The inexperienced and the unpractical again come forward with their childish and trivial objections, that artisans, small tradesmen, and townsmen generally cannot be converted into navvies, gardeners, and agricultural labourers. How do they know? It disgusts a practical man to hear such futile rubbish. Any man with legs and arms and a digestive apparatus can be taught and enabled to practise any calling, if the necessity be sharp enough. Even the members of the House of Lords—at any rate all those under sixty years of age—could be converted into average ploughmen in a few years with proper training. A high degree of civilisation seems to turn the majority of prosperous people into downright imbeciles. Does any reader of this article know anything of the farming districts of Yorkshire? If he does, he can testify that every year at harvest-time all the tailors, shoemakers, carpenters, and other journeymen and apprentice artisans, hire themselves out at a very high rate of wages to the neighbouring farmers for two, three, or four weeks. They go direct from their indoor occupations to the hardest possible kind of farm work; they get into full swing the very first day; they work long hours under a blazing sun; they have a change of diet as complete as can well be imagined —that is, from tea and bread, with meat once a day, to abundance of meat three times daily, and plenty of strong ale. What is the result? A general and most striking improvement in health in almost every instance. This is exactly in accordance with the experience gained in the Lancashire distress, except that the hardening process was a little longer in the case of the mill hands. What, however, did happen to the mill hands, who were converted by sharp necessity into navvies, was that they improved wonderfully in physique, and that many of them refused to return to the mills with returning prosperity, preferring to remain navvies to the end of their lives.

The points to be insisted upon are these: That in the Thames Purification there is a scheme of work ready to hand *now*, of such a kind as will effectually relieve the present distress; that if each one of the unemployed were taken in hand by a bonâ fide friend he might be at once put in the way of commencing to earn a livelihood. What stands in the way? Nothing but an unpractical, wealthy, and governing class. This scheme would not only give immediate and temporary relief; it would make town-dwellers over again. By converting them into diggers and navvies, it would fit them for Mr. Mills' Home Colonisation work; or, if that were not available, for emigration. In their present state, as has been said with wearisome iteration, they are both helpless and hopeless. Is not our scheme, at any rate, worth trying? Ought it not, in the absence of anything more perfect, to be tried? If a long season of severe cold should set in and drive the hungry to desperate deeds, it would be tried. Why not do, for the sake of pity and humanity, what everybody would rush madly to do if his skin or his pocket were in danger? As stated elsewhere, we propose to publish next week a distinct scheme for each constituency separately throughout the metropolis.

